# Combined meta-analysis of systemic effects of allogeneic stem cell transplantation and systemic sclerosis

**DOI:** 10.1186/2052-1839-14-7

**Published:** 2014-03-22

**Authors:** Dmitry N Grigoryev, Jignesh Dalal, Mara L Becker, Shui Q Ye

**Affiliations:** 1 Division of Experimental and Translational Genetics, Department of Pediatrics, Children’s Mercy Hospitals and Clinics, Kansas City, MO, USA; 2 Department of Biomedical and Health Informatics, Pediatric Research Building, Rm# 4729.02, University of Missouri School of Medicine, 2401 Gillham Road, Kansas City, MO, USA; 3 Division of Hematology Oncology, Department of Pediatrics, Children’s Mercy Hospitals and Clinics, Kansas City, MO, USA; 4 Division of Rheumatology, Department of Pediatrics, Children’s Mercy Hospitals and Clinics, Kansas City, MO, USA

**Keywords:** Meta-analysis, Allogeneic stem cell transplantation, Systemic sclerosis, Microarray, Molecular signature, Gene expression, Public data repository, Peripheral mononuclear cells, Circulating lymphocytes

## Abstract

**Background:**

Chronic graft-versus-host disease (cGVHD) is a major factor of morbidity and mortality for allogeneic stem cell transplantation (aSCT). The skin and internal organ involvement is the most common systemic complication of cGVHD and closely resembles systemic sclerosis (SSc). Circulating lymphocytes characterize the autoimmune nature of both conditions. Therefore we hypothesized that the common clinical manifestation (systemic organ and skin injury) and the common underlying players (lymphocytes) justify the combined meta-analysis of these diseases.

**Results:**

The aSCT and SSc datasets were uploaded from Gene Expression Omnibus (GEO), a public functional genomics data repository. The available microarray studies of peripheral blood mononuclear cells (PBMCs) and isolated lymphocytes were limited to well established microarray platforms (Affymetrix, Agilent, Canvac, and Illumina) and experimental settings with ≥10 patients per group. The resulting pools of data were merged by unique gene identifier and analyzed by the expression genome-wide association studies (eGWAS) coupled with the subtraction of the cGVHD^+^ and cGVHD^−^ molecular signatures. The eGWAS was applied to 47 and 50 lymphocyte profiles from aSCT and SSc patients, respectively. The identified 35 candidates were represented by 8 known cGVHD genes (including *CXCR4*, *LTBR* and *PML*) and 28 new candidate genes (including *SEPX1* and *DNJGB1*). The further mutual subtraction of cGVHD^+^ and cGVHD^−^ candidates and pathway analysis identified a list of 25 genes. Seven of these genes belong to the *fibroblast development and function* pathway, consisting of the well known cGVHD genes *CCND1*, *JUN*, and *FOS*, and the new molecular targets *MMP2*, *FOSB*, *TNFAIP8*, and *DUSP1*. These genes become primary candidates for a potential link of systemic effects of cGVHD and SSc.

**Conclusions:**

We designed a new approach for meta-analysis by combining data from different diseases using common clinical manifestation as a linker. This allowed us to power up the insufficient standalone meta-analysis of aSCT microarray studies, by adding SSc samples to the data pool. This new method has successfully identified novel molecular targets for systemic effects of both aSCT and SSc. We believe that this approach is generalizable and can be applied to an array of diseases with common clinical manifestations.

## Background

Allogeneic stem cell transplantation (aSCT) has become a curative therapy for increasing numbers of diseases. To date, it is the only successful cellular immunotherapy for high-risk malignancies such as leukemia. In pediatrics, it also offers curative therapy for nonmalignant blood disorders such as thalassemia, immune dysregulation, congenital bone marrow failure syndromes, inborn errors of metabolism and autoimmune conditions [1]. However, 30-70% of aSCT cases develop cGVHD, which occurs 100 days after transplantation with median time to onset of 4–6 months [2]. The precise mechanisms of cGVHD are unknown and evaluation of global transcriptional changes of reconstituted immune cells from aSCT patients is a feasible approach for studying cGVHD [3]. For instance, the transcript profiling of CD4^+^ and CD8^+^ lymphocytes from donors for aSCT demonstrated that predictive biomarkers for cGVHD can be detected [4].

Due to the paucity of microarray data for standalone meta-analysis of cGVHD, we used similarities in systemic responses between cGVHD and systemic sclerosis (SSc) to link their microarray datasets [5,6]. The similarity in systemic responses between cGVHD and SSc was demonstrated in the experimental settings and was attributed to the excessive activation of T and B cells in both conditions [7].Therefore, we hypothesized that the combined meta-analysis of both diseases would lead to better understanding of systemic effects of circulating lymphocytes in aSCT and SSc.

In this study we combined publicly available datasets for aSCT (with or without cGVHD) and SSc (diffuse and limited) and analyzed them using a meta-analysis technique.

## Results and discussion

**Figure 1:**
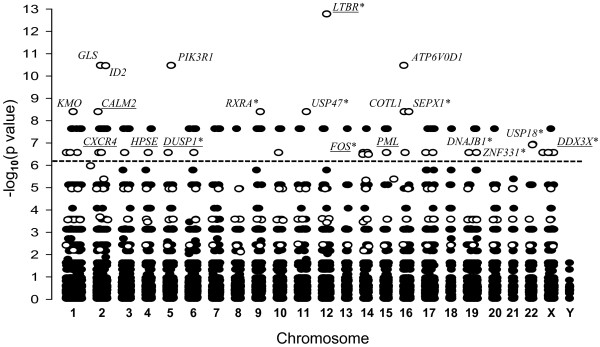
**eGWAS for aSCT and SSc using a *χ***^**2 **^**analysis.** The significance, P values, of each tested gene is expressed as − log_10_ (*y axis*) and plotted against corresponding chromosomal location (*x axis*). P values for each gene were calculated for 7 microarray datasets (4 aSCT and 3 SSc) as the likelihood of finding repeated differential expression compared with expected using *χ*^*2*^ analysis. Out of 32671 genes tested 118 genes demonstrated a significant differential expression, of which 101 had the known chromosomal location. The dotted line indicates the Bonferroni threshold (P = 1.53 × 10^−6^). The gene symbols indicate genes that are most significantly associated with both clinical aSCT and SSc. The open circles represent genes that demonstrated significant changes in both diseases and the solid circles represent genes with significant changes only in one of tested diseases. Underscored symbols indicate known cGVHD genes. * - genes that were also detected by subtraction of ranking order gene lists.

A total of 32671 genes were analyzed by eGWAS and 35 genes were identified as associated with both diseases, while 77 genes were associated with SSc only. Genes associated with aSCT alone were not detected (Figure 1 and Additional file 1) due to the low statistical power of cGVHD data. These findings support our approach and provided 35 potential candidates, which otherwise would have been missed by conventional analysis.

Eight genes among these candidates were previously linked to cGVHD (Figure 1), which demonstrated that despite the amplification of cGVHD data with unrelated but similar clinical conditions, we generated a feasible candidate list for cGVHD. The top candidate was lymphotoxin beta receptor or TNFR superfamily, member 3 (*LTBR*) with P = 1.73 × 10^−13^ (Figure 1). Concordantly, it was recently reported that the expression of *LTBR* is elevated in patients with cGVHD^+^ as compared to cGVHD^−^, and that the application of lymphotoxin beta receptor-immunoglobulin fusion protein in mouse model of cGVHD is beneficial [8,9].

Given that eGWAS confirmed the feasibility of combined meta-analysis of two different diseases with common clinical manifestations, we proceeded with the mutual subtraction of molecular signatures of cGVHD and SSc.

**Figure 2:**
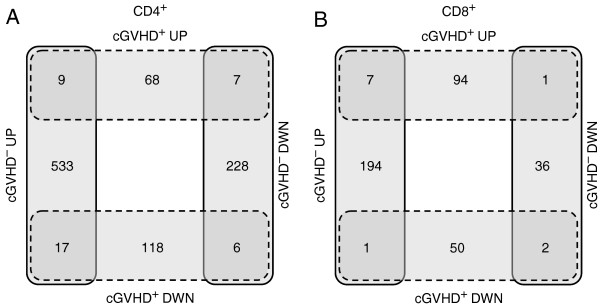
**Detection of cGVHD specific transcripts in CD4**^**+ **^**and CD8**^**+ **^**lymphocytes.** Transcripts that were up- or down- regulated in control population of aSCT patients without cGVHD were subtracted from transcripts that were up- or down- regulated in patients with cGVHD. The subtraction was conducted by cross-referencing of transcriptional changes in CD4^+^ (*panel ****A***) or CD8^+^ (*panel ****B***) lymphocytes, respectively. Transcripts that are common to lymphocytes from control and cGVHD subjects were eliminated from further consideration.

We began with stratified analysis of CD4^+^ and CD8^+^ T-cells from patients with cGVHD and identified 225 CD4^+^ and 155 CD8^+^ transcripts significantly affected by the new host environment. The analysis of CD4^+^ and CD8^+^ T-cells from patients who underwent aSCT and remained cGVHD^−^ one year later identified 800 and 241 transcripts with significant changes, respectively. 84 up- and 141 down-regulated cGVHD^+^ transcripts of CD4^+^ lymphocytes were cross-referenced against 559 up- and 241 down-regulated aSCT transcripts (Figure 2A). This approach identified 68 up- and 118 down-regulated transcripts that were specific for cGVHD^+^. In addition, 24 transcripts were oppositely regulated in cGVHD^+^ and cGVHD^−^ CD4^+^ cells: 7 were up-regulated in cGVHD^+^, but down-regulated in cGVHD^−^ cells, and 17 were down-regulated in cGVHD^+^, but up-regulated in cGVHD^−^ cells (Figure 2A), which add up to 210 cGVHD^+^ specific transcripts in CD4^+^ cells. The similar cross-referencing of CD8^+^ T-lymphocytes identified 94 up- and 50 down-regulated transcripts that were specific for cGVHD^+^. In addition, 2 transcripts were oppositely regulated in cGVHD^+^ and cGVHD^−^ CD8^+^ cells: one was up-regulated in cGVHD^+^, but down-regulated in cGVHD^−^ cells, and another was down-regulated in cGVHD^+^, but up-regulated in cGVHD^−^ cells (Figure 2B), which were added up to 146 cGVHD^+^ specific transcripts in CD8^+^ cells.

The comparison of molecular changes of CD4^+^ and CD8^+^ T-lymphocytes 1 year after aSCT was conducted by chi-square test and demonstrated a significant difference (P < 0.0001) in ratios of up- and down-regulated genes between these cell types. In CD4^+^ T-cells the molecular signature was shifted towards suppression of gene expression (75 up-regulated genes vs 135 down-regulated genes), while in CD8^+^ T-cells the molecular signature was shifted towards activation (95 up-regulated genes vs 51 down-regulated genes). The overall transcriptional changes were more pronounced in CD4^+^ cells (210 genes) versus CD8^+^ cells (146 genes). These findings suggested that CD4^+^ plays a bigger role in aSCT than CD8^+^.

**Figure 3:**
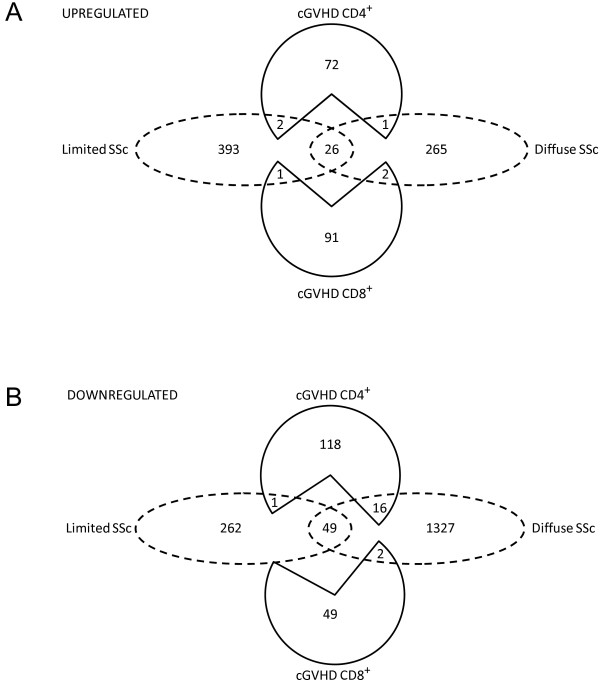
**Cross-referencing cGVHD gene candidates with molecular signature of SSc.** CD4^+^ (*top*) and CD8^+^ (*bottom*) lymphocytes were cross-referenced against limited SSc or diffuse SSc, respectively. Transcripts that were upregulated (*panel ****A***) or downregulated (*panel ****B***) in both cGVHD and scleroderma patients were considered to be associated with the skin manifestation of cGVHD.

The cross-referencing transcripts associated with cGVHD against transcripts associated with limited or diffuse SSc identified 6 up-regulated genes (Figure 3A) and 19 down-regulated genes (Figure 3B) that were common to both diseases, of which 4 genes were involved in limited SSc and 21 were associated with diffuse SSc.

The significance of co-expression of these 25 genes was evaluated by Student’s T-test. The contribution of CD4^+^ lymphocytes to both cGVHD and SSc was 2.55 times higher (P = 0.033) than the contribution of CD8^+^ lymphocytes, which confirmed our observation of more pronounced involvement of CD4^+^. We next tested which type of SSc the molecular signature of cGVHD lymphocytes most resembles. While we were able to demonstrate that the molecular signature of cGVHD lymphocytes resembles diffuse SSc by 2.2 fold more than limited SSc, the t-test of this comparison did not reach significance (p = 0.124).

The greater contribution of CD4^+^ to cGVHD molecular signature was further confirmed by the pathway analysis. We evaluated the relevance of cGVHD transcripts to known biological processes using the Ingenuity Pathway Analysis (IPA) tool (http://www.ingenuity.com). 163 out of 210 transcripts of CD4^+^ lymphocytes and 127 out of 146 transcripts of CD8^+^ lymphocytes were mapped by the software to known genes, while the unmapped transcripts consisted of hypothetical proteins and open reading frames.

**Figure 4:**
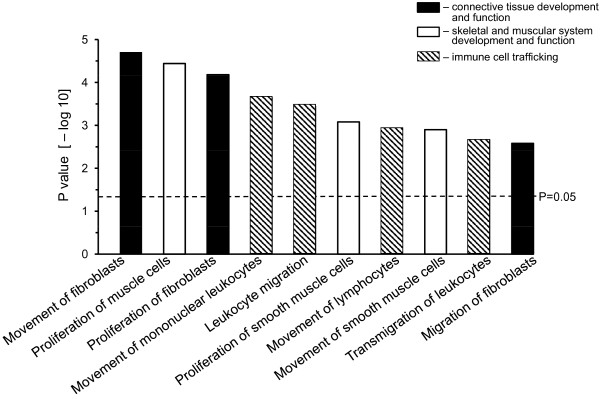
**Pathway analysis of cGVHD specific transcripts in CD4**^**+ **^**lymphocytes.** 210 cGVHD transcripts were analyzed by the Ingenuity Pathway Analysis (IPA) software. IPA mapped 163 of these transcripts to known genes and identified three major processes associated with cGVHD: *connective tissue development and function* (solid bars), *skeletal and muscular system development function* (open bars), and *immune cell trafficking* (hatched bars). The height of each bar represents − log_10_P value (*y axis*) of corresponding biological function (*x axis*). The dashed line indicates the P value = 0.05 (−log_10_P = 1.301).

The top three bioprocesses identified by IPA for CD4^+^ were *connective tissue development and function*, *skeletal and muscular system development and function*, and *immune cell trafficking*. These bioprocesses were built of multiple functional subprocesses, where *movement of fibroblasts* was the most significant function (Figure 4). None of the top three bioprocesses identified by IPA for CD8^+^ contained 5 or more genes, and therefore did not pass filtering criteria.

**Figure 5:**
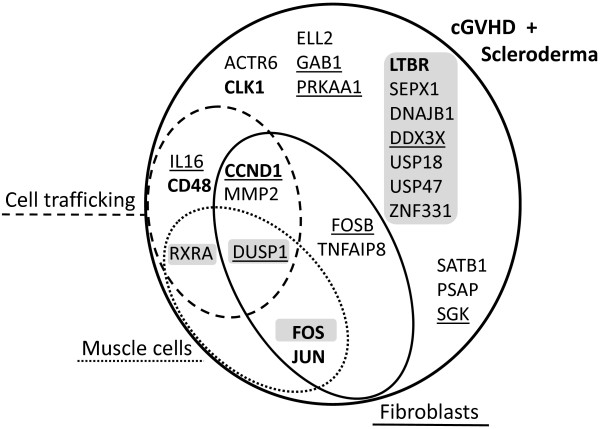
**Pathway distribution of cGVHD-SSc gene candidates.** The detailed output of pathway analysis was queried against 25 gene candidates (main circle), which are potentially associated with the skin manifestation of cGVHD. Genes were grouped according to involvement in three major pathways (Figure 5): *connective tissue development and function* (solid oval), *skeletal and muscular system development function* (dotted oval), and *immune cell trafficking* (dashed oval). The known cGVHD genes are bolded. The candidates, which were reported in the original aSCT study, are underscored. Genes that were identified by our eGWAS studies (Figures 1) are highlighted by grey boxes.

The top three IPA pathways were overrepresented by fibroblast related genes (7 genes). The immune cell trafficking (5 genes), and muscle cell related functions (4 genes) (Figure 5). While 8 out of 25 candidates that we have detected were reported by the original studies uploaded from GEO, the remaining 17 candidates appeared to be missed as potential cGVHD candidates (Figure 5).

These genes were unreported by the original studies, most likely due to different goals and focuses of the authors. This is an example of how the re-analysis of publicly available data using new approaches will bring to light molecular targets unsuspected by the original studies.

**Figure 6:**
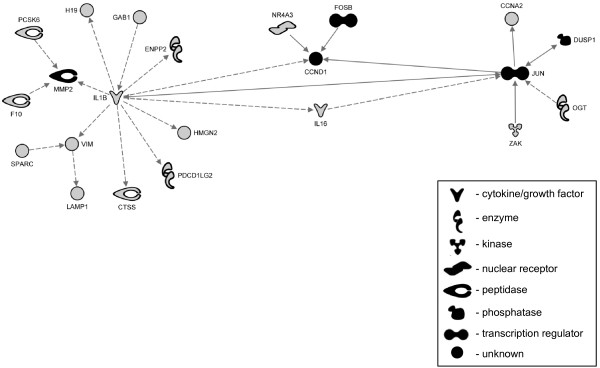
**The IPA network analysis of cGVHD-SSc gene candidates.** Several networks were generated for 163 cGVHD candidate genes significantly affected by new host environment in CD4^+^ lymphocytes. The top network is presented here and is limited to genes with direct relationships with candidates for the skin manifestation of cGVHD. Relationships between genes are represented by solid lines (binding), solid arrows (direct activation), or broken arrows (indirect activation). Arrows point to the element on which an action is performed. Black molecules represent fibroblast related genes. Grey molecules represent other significant cGVHD genes.

The same group of 25 genes was used for filtering biological networks generated by IPA software. The resulting network represented two major nodes built of *IL1B* and *JUN* related molecular groups that were cross-linked by *FOSB*-*CCND1* node of fibrotic candidate genes (Figure 6). The existing interactions between these gene candidates suggest their common involvement in the fibrotic response during cGVHD, thus further validating our target selection.

PubMatrix analysis of 25 gene candidates common to cGVHD and SSc identified 7 genes that were previously linked to GVHD (Table 1). The analysis of mutually subtracted cGVHD^+^ and cGVHD^−^ genes identified 6 genes that were previously linked to GVHD, 3 of which were linked to the fibroblast proliferation bioprocess (Table 1). This suggests that our new genes identified by these approaches will be also valid candidates for cGVHD. Moreover, despite the completely different approaches between eGWAS and cross-referencing the molecular signatures of cGVHD and SSc, 10 genes out of 25 detected by cross-referencing (Figure 5) were also among 35 genes detected by eGWAS (Figure 1).

**Table 1:** Gene candidates for skin manifestation of cGVHD

**Gene**	**Symbol**	**Gene**	**Lympho-cyte**	**Stem cell transplantation**	**Graft versus host disease**	**Systemic sclerosis**
Jun proto-oncogene*	**JUN**	**13683**	**1546**	**83**	**16**	**33**
Lymphotoxin beta receptor	LTBR	94	93	6	9	0
CD48 molecule	CD48	136	275	29	4	0
**Cyclin D1**	**CCND1**	**7324**	**424**	**76**	**3**	**1**
**FBJ murine osteosarcoma viral oncogene homolog**	**FOS**	**15253**	**836**	**31**	**1**	**10**
Interleukin 16	IL16	129	242	2	1	1
CDC-like kinase 1	CLK1	27	0	1	1	0
**Matrix metallopeptidase 2 (gelatinase A)**	**MMP2**	**906**	**45**	**30**	**0**	**4**
DEAD (Asp-Glu-Ala-Asp) box polypeptide 3, X-linked	DDX3X	57	2	2	0	0
GRB2-associated binding protein 1	GAB1	114	23	1	0	0
**FBJ murine osteosarcoma viral oncogene homolog B**	**FOSB**	**522**	**22**	**1**	**0**	**0**
**Tumor necrosis factor, alpha-induced protein 8**	**TNFAIP8**	**14**	**4**	**1**	**0**	**0**
Prosaposin	PSAP	178	11	0	0	1
SATB homeobox 1	SATB1	146	61	0	0	0
**Dual specificity phosphatase 1**	**DUSP1**	**392**	**18**	**0**	**0**	**0**
Ubiquitin specific peptidase 18	USP18	71	7	0	0	0
Dnaj (Hsp40) homolog, subfamily B, member 1	DNAJB1	67	7	0	0	0
Elongation factor, RNA polymerase II, 2	ELL2	22	6	0	0	0
Serum/glucocorticoid regulated kinase	SGK	211	3	0	0	0
Retinoid X receptor, alpha	RXRA	77	3	0	0	0
Zinc finger protein 331	ZNF331	18	2	0	0	0
Protein kinase, AMP-activated, alpha 1 catalytic subunit	PRKAA1	77	1	0	0	0
Methionine sulfoxide reductase B1	SEPX1	5	1	0	0	0
ARP6 actin-related protein 6 homolog (yeast)	ACTR6	1	0	0	0	0
Ubiquitin specific peptidase 47	USP47	1	0	0	0	0

*Bolded are genes related to fibroblast proliferation.

Of the remaining genes considered new candidates for cGVHD, MMP2, FOSB, TNFAIP8, and DUSP1 were from the fibroblast proliferation pathway and all, but TNFAIP8, were assigned to the same gene-gene interaction network (Figure 6). Moreover, amongst these genes, *DUSP1* was detected by eGWAS studies as well (Figure 1), therefore *DUSP1* became our primary candidate.

The expression of *DUSP1* in lymphocytes had been detected by many groups (18 citations), however, this gene has not yet been implicated in aSCT or SSc (Table 1). Despite this, the recent studies of circulating cells in peripheral blood of kidney transplant patients identified *DUSP1* as a potential biomarker for monitoring renal graft status [10]. We believe that results of our meta-analysis and the reported allograft sensitivity of *DUSP1* warrants further studies of this gene in the cGVHD setting.

The other new candidate, *MMP2*, had the strongest link to aSCT (30 citations) and was detected in scleroderma (2 citations). However it has not yet been linked to cGVHD (Table 1 and Figure 5). In addition to its ability to degrade extracellular matrix proteins, MMP2 can also act on several nonmatrix proteins such as calcitonin gene-related peptide, thus promoting vasoconstriction. Moreover, its C-terminal non-catalytic fragment possesses an anti-angiogenic property (http://www.uniprot.org). One can speculate that the combination of vasoconstriction and anti-angiogenisity can be contributory to hypoxia and systemic sclerosis. Furthermore, the potential immunogenetic role of MMP2 was observed in a mouse model of heart transplant. It was demonstrated that alloreactivity of T-cells was significantly lower in MMP2-deficient mice compared to their wild type littermates [11]. All these findings make MMP2 an extremely appealing target for cGVHD.

Our final candidate, FOSB, is less known in aSCT (1 citation), but was reported in supplemental materials of original studies submitted to GEO [4]. The gene-gene interaction analysis put this gene into the central cross-linker nodule of two major nodes of cGVHD candidates (Figure 6). The main function of FOSB is believed to enhance binding activity of JUN proteins to DNA. Furthermore, the finding that during induced clonal anergy of CD4^+^ T-cells, JUN and FOS proteins, but not FOSB, reduce their DNA-binding ability [12] makes FOSB an interesting candidate that may prolong the active state of T lymphocytes under suppressive conditions, thus promoting the aSCT complications.

## Conclusions

Presented here data indicate that: 1) for the first time in the field of meta-analysis of microarray data we conducted cross-disease analysis based on common clinical manifestations; 2) CD4^+^ T-cells undergo more pronounced changes during cGVHD than their CD8^+^ counterparts and these changes are shifted towards down-regulation; 3) the molecular signature of cGVHD does not demonstrate preferable similarity with either type of systemic sclerosis; 4) the pathway analysis linked expressional changes in lymphocytes from cGVHD patients to fibroblast proliferation; 5) we identified 25 gene candidates common to both cGVHD and SSc, of which primary candidates MMP2, FOSB, and DUSP1are the most appealing for further studies. We believe that our approach of *in silico* amplification of sparse microarray data by linking molecular signatures of different diseases with the same systemic complications and conducting meta-analysis on such combined dataset is generalizable and capable of detecting new molecular targets.

## Methods

### Data

The aSCT and SSc datasets were uploaded from gene expression omnibus (GEO, NCBI, http://www.ncbi.nlm.nih.gov/geo). The term “bone marrow transplant” was submitted to the database query windows and this search returned 81 entries (as of December 14, 2012), 23 of which were data series, while the remaining data were either annotation of microarray platforms or individual samples.

The inclusion criteria for retrieved data were the array samples, which must: 1) represent genome wide studies of human peripheral blood mononuclear cells (PBMCs) or isolated lymphocytes; 2) be generated with established microarray platforms and the number of interrogated sequences should be >5000, thus excluding custom platforms that are pathway oriented and will introduce bias towards given biological process; and 3) the experimental settings must have ≥10 patients per condition.

The studies that satisfied these criteria are listed in Table 2. We utilized data from 5 different experimental settings, which were generated using 4 different microarray platforms. The specific GEO sample IDs (GSM), which were used for this study and a brief description of the experimental settings are listed below:

**Table 2:** Summary of microarray data used for meta-analysis

**Condition**	**Cell type**	**Healthy cells**	**Diseased cells**	**GEO ID**	**Microarray platform**	**Author**
aSCT	CD4/CD8	12/12	10/10	GSE4624	Canvac	Baron et al [4].
aSCT + cGVHD	CD4/CD8	18/17	14/13	GSE4624	Canvac	Baron et al. [4]
Diffuse SSc	PBMCs	41	19	GSE33463	Illumina	Cheadle et al. [13]
Diffuse SSc	PBMCs	10	10	GSE22356	Affymetrix	Risbano et al. [14]
Limited SSc	PBMCs	10	21	GSE19617	Agilent	Pendergrass et al. [15]

Baron *et al*. [4]: GSM103566-67, GSM103610-11, GSM103616-17, GSM103642-43, GSM103680-81, GSM103684-85, GSM103700-01, GSM103712-13, GSM103716-17, GSM103720-21, GSM103724-25, and GSM103728-29. Their CD4^+^/CD8^+^ cells were from 12 donors, whose cells did not cause cGVHD in a recipient 1 year after aSCT; GSM103626-27, GSM103630-31, GSM103634-35, GSM103638-39, GSM103650-51, GSM103668-69, GSM103678-79, GSM103686-87, GSM103689, GSM103696-97, GSM103568-69, GSM103570-71, GSM103573, GSM103584-85, GSM103592-93, GSM103594-95, GSM103604-05, GSM103704-05, and GSM103707. Their CD4^+^/CD8^+^ cells were from 18 donors, whose cells did cause cGVHD in a recipient 1 year after aSCT (one CD8^+^ sample was not reported, see Table 2); GSM103620-21, GSM103640-41, GSM103710-11, GSM103714-15, GSM103718-19, GSM103722-23, GSM103726-27, GSM103652-53, GSM103682-83, and GSM103698-99. Their CD4^+^/CD8^+^ cells were from 10 aSCT patients, who remain cGVHD-free 1 year after aSCT; GSM103622-23, GSM103624-25, GSM103628-29, GSM103632-33, GSM103636-37, GSM103572, GSM103582-83, GSM103590-91, GSM103618-19, GSM103648-49, GSM103666-67, GSM103688, GSM103694-95, GSM103702-03, and GSM103706. Their CD4^+^/CD8^+^ cells were from 14 cGVHD patients, who developed cGVHD 1 year after aSCT (one CD8^+^ sample is not reported, see Table 2).

Cheadle *et al*. [13]: GSM827665-705 – PBMCs were from 41 healthy controls, and GSM827778-96, PBMCs from 19 patients with diffuse SSc.

Risbano *et al*. [14]: GSM556441-50, PBMCs were from 10 healthy controls, and GSM556413-22, PBMCs from 10 patients with diffuse SSc.

Pendergrass *et al.*[15]: GSM489236-41, GSM489243, GSM489245-47, PBMCs were from 10 healthy controls, and GSM489194-98, GSM489200-01, GSM489204, GSM489207-218, PBMCs from 21 patients with limited SSc.

#### Linking different microarray platforms

Obtained GSMs were cross-linked based on the GEO provided Minimum Information About a Microarray Experiment (MIAME) data as described previously [16,17]. Briefly, the probe IDs across different microarray platforms were linked using the Array Information Library Universal Navigator (AILUN) tool (http://ailun.stanford.edu) and the probes that remained unmatched by AILUN were added to the main AILUN created dataset by matching HUGO-approved gene symbols.

### Gene candidate selection

To estimate differences between groups of samples from aSCT and aSCT + cGVHD patients, and healthy and SSc patients, raw post-quantitation microarray data was median trimmed using minimal trim of 1 highest and 1 lowest expressors. Given the general trend of microarray data series to have low number of biological replicates, the more vigorous trimming was not applied, due to the great reduction of statistical power. The probes without a positive signal throughout all the experiments were then removed, and the remaining data were reanalyzed using SAM 2.0 software [18], applying default settings without application of arbitrary restrictions [19], as described previously [20]. For each gene in every microarray experiment, the *d* score, which denotes the standardized change in gene expression of diseased lymphocytes versus corresponding healthy lymphocytes, was calculated. Given that the total of 32671 probes were analyzed, the Bonferroni threshold (P = 1.53 × 10^−6^) was used for detecting significance.

For eGWAS analysis, 32671 probes from all experiments were merged into one dataset and significant and not significant changes counted and [4] evaluated by chi-square test as described previously [21]. Genes with an absolute value of *d* score ≥2 [17] or an absolute value of fold change ≥1.2 [22] between healthy and diseased groups were considered significantly dysregulated.

### Molecular signature subtraction

The gene lists for subtraction analysis were generated using “ranking order” approach. SAM output was ranked according to the fold change and genes from the top quartile were considered significantly affected by a corresponding disease. The subtraction of transcript lists was conducted using VENNY tool (http://bioinfogp.cnb.csic.es/tools/venny/index.html). The significance of association of lymphocyte types (CD4^+^ or CD8^+^) in cGVHD samples with the PBMC samples from scleroderma types (diffuse or limited) was assessed using two tailed T-test. Given that scleroderma is represented in the GEO collection by PBMC data only, but knowing that PBMCs are comprised of up to 60% of CD4^+^ and up to 30% of CD8^+^, we considered it acceptable to directly compare CD4^+^ and CD8^+^ fractions of cGVHD samples with PBMC samples of SSc patients. Values submitted to the T-test were first normalized to the total number of lymphocyte type or scleroderma type, respectively. Association between molecular signatures with P value <0.05 was considered significant.

### Automated literature search

PubMatrix (multiplex literature mining tool) analysis [23] was conducted as described previously [22]. We restricted our search to human symbols approved by HUGO Gene Nomenclature Committee (HGNC), which were enriched by all aliases and former (discontinued) symbols for selected candidate genes (http://www.genenames.org). The symbols with the hyphen and double names separated by a slash were filtered out. The biomedical literature reference count for a given gene was represented by the highest number among HUGO symbol or its aliases. The genes with number of references N ≥ 5 were considered to have established association with cGVHD, 5 > N > 0 were considered novel (have been noticed during cGVHD studies, but were not linked to the disease), and N = 0 were considered new.

### Pathway analysis

The pathway analysis, which links the most relevant biological processes to a provided list of candidate genes, was conducted using the Ingenuity Pathways Knowledge Base tool (Ingenuity Systems, Inc., Redwood City, CA.) as described previously [24,25]. Gene symbols from CD4^+^ and CD8^+^ significant gene lists were submitted to the IPA and analyzed using “Core” tool. Significance of the identified pathways was tested by the IPA imbedded Fisher Exact test and expressed as p-value [26]. Pathways, that were built of 5 or more genes and had p < 0.05 were considered significant.

## Abbreviations

AILUN: Array Information Library Universal Navigator; aSCT: Allogeneic stem cell transplantation; cGVHD: Chronic graft-versus-host disease; eGWAS: Expression genome-wide association studies; GEO: Gene expression omnibus; GSM: GEO sample IDs or gene expression series matrix; HGNC: HUGO Gene Nomenclature Committee; IPA: Ingenuity pathways analysis; LTBR: Lymphotoxin beta receptor or TNFR superfamily, member 3; MIAME: Minimum information about a microarray experiment; NCBI: National Center for Biotechnology Information; PBMCs: Peripheral blood mononuclear cells; SSc: Systemic sclerosis.

## Competing interest

The authors declare that they have no competing interests.

## Authors’ contributions

DNG, JD and SQY conceived of the study. MLB participated in its design, JD and MLB carried out clinical sample selection from the publicly available data. DNG and SQY did statistical analyses of all data and drafted the manuscript. All authors read and approved the final manuscript.

## Pre-publication history

The pre-publication history for this paper can be accessed here:

http://www.biomedcentral.com/2052-1839/14/7/prepub

## Supplementary Material

Additional file 1 Supplemental Table 1. Click here for file
